# Quaternary climatic fluctuations and resulting climatically suitable areas for Eurasian owlets

**DOI:** 10.1002/ece3.5086

**Published:** 2019-03-26

**Authors:** Pankaj Koparde, Prachi Mehta, Shomita Mukherjee, V. V. Robin

**Affiliations:** ^1^ Division of Conservation Biology Sálim Ali Centre for Ornithology and Natural History Coimbatore India; ^2^ Manipal Academy of Higher Education Manipal India; ^3^ Indian Institute of Science Education and Research (IISER) Tirupati India; ^4^ Wildlife Research and Conservation Society Pune India

**Keywords:** citizen science, comparative biogeography, Forest Owlet, geographical range, last glacial maximum, little owl, quaternary climatic fluctuations

## Abstract

**Aim:**

The nested pattern in the geographical distribution of three Indian owlets, resulting in a gradient of endemicity, is hypothesized to be an impact of historical climate change. In current time, the Forest Owlet *Athene blewitti* is endemic to central India, and its range is encompassed within the ranges of the Jungle Owlet *Glaucidium radiatum *(distributed through South Asia) and Spotted Owlet *Athene brama* (distributed through Iran, South and Southeast Asia). Another phylogenetically close species, Little Owl *Athene noctua, *which is largely Palearctic in distribution, is hypothesized to have undergone severe range reduction during the Last Glacial Maximum, showing a postglacial expansion. The present study tests hypotheses on the possible role of Quaternary climatic fluctuations in shaping geographical ranges of owlets.

**Methods:**

We used primary field observations, open access data, and climatic niche modeling to construct climatic niches of four owlets for four periods, the Last Interglacial (~120–140 Ka), Last Glacial Maximum (~22 Ka), Mid‐Holocene (~6 Ka), and Current (1960–1990). We performed climatic niche extent, breadth, and overlap analyses and tested if climatically suitable areas for owlets are nested in a relatively stable climate.

**Results:**

Climatically suitable areas for all owlets examined underwent cycles of expansion and reduction or a gradual expansion or reduction since the Last Interglacial. The Indian owlets show significant climatic niche overlap in the current period. Climatically suitable areas for Little Owl shifted southwards during the Last Glacial Maximum and expanded northwards in the postglaciation period. For each owlet, the modeled climatic niches were nested in climatically stable areas.

**Main Conclusions:**

The study highlights the impact of Quaternary climate change in shaping the present distribution of owlets. This is relevant to the current scenario of climate change and global warming and can help inform conservation strategies, especially for the extremely range‐restricted Forest Owlet.

## 1 INTRODUCTION

Past climatic fluctuations have played a major role in shaping the ranges of several species, especially endemic and endangered species in the regions harboring much of today's biodiversity such as the tropics (Bose, Munoz, Ramesh, & Pélissier, 2016; Bueno et al., 2017; Carnaval & Moritz, 2008; Costa et al., 2018; Pinilla‐Buitrago, Escalante, Gutiérrez‐Velázquez, Reyes‐Castillo, & Rojas‐Soto, 2018; Werneck, Nogueira, Colli, Sites, & Costa, 2012). Regions that experience low climatic variation (climatically stable) are believed to have more endemic species (Dynesius & Jansson, 2000). Jansson (2003) proposed that Milankovitch cycles during the Quaternary are responsible for current global geographical patterns of endemic species. The effect of the Last Interglacial (LIG: ~120 –140 Ka), Last Glacial Maximum (LGM: ~18–22 Ka), and Mid‐Holocene (MDH: ~6 Ka) seems to be very prominent for several taxa (Jansson, 2003; Ramachandran, Robin, Tamma, & Ramakrishnan, 2017). Here, both the LIG and LGM periods represent periods of extreme climatic conditions.

During the LIG, temperatures warmer than the pre‐industrial Holocene climate prevailed globally (*reviewed in *Kukla et al., 2002; Otto‐Bliesner, Marshall, Overpeck, Miller, & Hu, 2006), with tropical areas exhibiting robust monsoonal systems (Pedersen, Langen, & Vinther, 2017). The LGM was the most recent driest period on Earth. The LGM characterized low average temperature, increased aridity, and a drop in sea levels (Clark & Huybers, 2009), leading to a change in climate, available land area, and climate‐associated changes in vegetation (Anhuf et al., 2006; Bose et al., 2016). These changes possibly altered the ranges of many species. In the Holocene (~11.7 Ka to Present), a warmer climate than the LGM prevailed in the Northern Hemisphere but the tropics were colder than the present (Mayewski et al., 2004; Steig, 1999; Wanner et al., 2008).

Knowledge from the Quaternary period suggests that species responses to past climate change can provide crucial information on their current and future evolutionary and ecological trajectories. Birds are among the most widely studied taxa with respect to climate change effects. Studies have shown a drastic negative impact of climate change on bird distributions (Hilbert, Bradford, Parker, & Westcott, 2004; Ramachandran et al., 2017; Smith, Gregory, Anderson, & Thomas, 2013) and demography (Howard et al., 2018; Tomotani et al., 2018; Zhao et al., 2012), in many cases compromising their persistence (Crick, 2004; Urban, 2015).

In this paper, we examine the effect of the Quaternary climatic fluctuations on the climatic niche extents of owlets that show a gradient of endemicity and overlap in their current geographical distributions in parts of their ranges. Such comparative biogeography studies are scarce and have been recommended to comprehend community responses to global climate change (Berg et al., 2010). Six species of owlets are known from India, of which Jungle Owlet *Glaucidium radiatum *(Temminck), Spotted Owlet *Athene brama *(Temminck), and the highly range‐restricted and Endangered Forest Owlet *Athene blewitti *(Hume) (BirdLife International, 2017) are sympatric in central India. The Little Owl *Athene noctua *(Scopoli) of Palearctic region partially overlaps in distribution with the Spotted Owlet. The four owlets with varying range extents, habitat requirements and degrees of overlap with each other are phylogenetically closely related (Koparde et al., 2018). Understanding how their ecological and evolutionary histories shaped their current distribution can provide vital information on how they respond to climatic changes, which will help plan their conservation strategies in the current scenario, especially in case of the Endangered Forest Owlet.

Divergence estimates from the phylogeny of Indian owlets indicate that the Plio‐Pleistocene climate change may have played an important role in the speciation of *Athene *and *Glaucidium *owlets (Koparde et al., 2018) that could explain patterns of their current ranges. Pellegrino et al., (2014), suggest that the Little Owl survived in the European Southern Refugia (Iberian, Italian, and Balkan Peninsula) during the LGM, when much of its distributional range was covered in ice, and later expanded into its current range.

In the present study, we explore if Quaternary climatic fluctuations played a role in shaping the geographical distributions of owlets, using past‐projected climatic niche models (CNMs) and examine if the suitable areas for the endemic and Endangered Forest Owlet were nested within climatically stable areas to a greater extent, as compared to the other relatively widespread species.

## 2 METHODS

### 2.1 Target species

**Figure 1:**
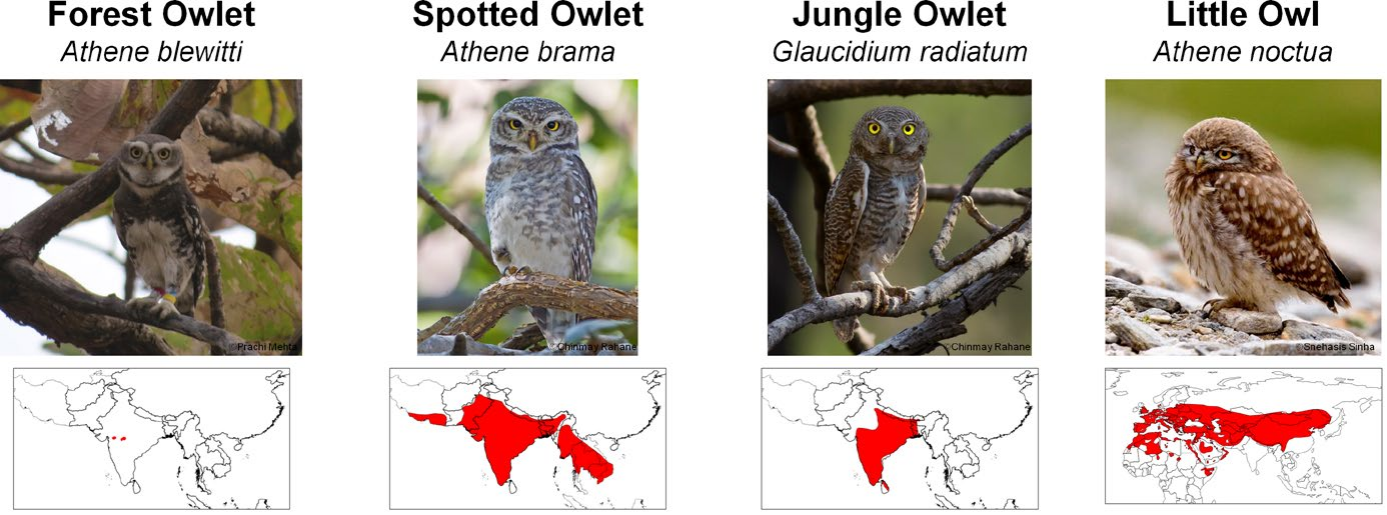
The study owlets and their present distribution provided by Birdlife International (2016a, 2016b, 2016c, 2017). Red regions indicate the geographical distribution of species. Photo credits: Forest Owlet by PM, Spotted Owlet and Jungle Owlet by Chinmay Rahane, and Little Owl by Snehasis Sinha

The four target species are the Forest Owlet, Jungle Owlet, Spotted Owlet, and Little Owl and are presented in Figure 1. The Forest Owlet has a narrow and severely fragmented range across central India with an Extent of Occurrence (EOO) of 55,300 km^2 ^(Birdlife International, 2017) and hence is a priority species in conservation. The Jungle Owlet occurs across Peninsular India and Sri Lanka (EOO = 3,470,000 km^2^) (Birdlife International, 2016a). The Spotted Owlet is distributed across most of the Indian Subcontinent, from Iran to the West to Myanmar in the East and from the Himalayas in the North to the Southern tip of India, except in Sri Lanka (EOO = 10,800,000 km^2^) (Birdlife International, 2016b). The three owlets (henceforth referred as “Indian owlets”) show a gradient of endemicity and a nested pattern in the extent of their geographic ranges. The Little Owl, sister to Spotted Owlet, mainly distributed in the Palearctic (EOO = 52,900,000 km^2^) (Birdlife International, 2016c), also occurs in the Indian Himalayas, partially overlapping in geographical distribution with the Spotted Owlet. Since the owlets are of similar size (20–24 cm) (Ali & Ripley, 1983; Rasmussen & Collar, 1998) and Forest Owlet distribution overlaps completely with Spotted Owlet and Jungle Owlet distributions in central India, they can be potential competitors in areas of sympatry (Ishtiaq, 2000; Jathar & Rahmani, 2004; Mehta, Kulkarni, Talmale, & Chandarana, 2018; Rasmussen & Ishtiaq, 1999). At a finer scale, however, their habitat associations are reported to be different. The Spotted Owlet and Little Owl are associated with open habitats and considered to be synanthropic while the Jungle Owlet is associated with dry to moist deciduous open forests and scrublands (Ali & Ripley, 1983). The Forest Owlet is restricted to the Teak dominated dry deciduous forests of central India (Mehta, Kulkarni, Mukherjee, Chavan, & Anand, 2017; Mehta, Kulkarni, Patil, Kolte, & Khatavkar, 2008).

We obtained Forest Owlet locations from published literature (*n* = 30) (Chavan & Rithe, 2009; Ishtiaq & Rahmani, 2000,2005; Jathar & Rahmani, 2004; Kasambe, Pande, Wadatkar, & Pawashe, 2004; King & Rasmussen, 1998; Laad & Dagale, 2014; Patel et al., 2015) and primary field observations (*n* = 25). We filtered the initial dataset of 55 points to 50 points, avoiding spatially overlapping points. We did not use citizen science based portals for collecting Forest Owlet location data, due to uncertainty associated with these reports and the accuracy of location coordinates. For other owlets, we collected presence locations from eBird (eBird, 2017; Sullivan et al., 2009) and iNaturalist (iNaturalist, 2017). The eBird and iNaturalist observations were filtered to restrict the duration to the years from 1970 to 2016 and include coordinate certainty below 2 km. We curated the point locations for all other owlets and filtered them by country (excluding countries where the species is introduced or traded), date (including records from 1970 to 2016), area (including GPS coordinate certainty below 2 km), and approved and reviewed status. We retrieved a total of 23,243 and 202 points for Little Owl from eBird and iNaturalist, respectively. By using the country filter, points from New Zealand were avoided, where the species has been introduced recently. Finally, 2,438 and 84 points (total *n* = 2,522) were retained from eBird and iNaturalist, respectively. Similarly, we filtered 18,472 eBird and 73 iNaturalist records of Spotted Owlet to a final count of 6,042 eBird and 27 iNaturalist records (total *n* = 6,069). For Jungle Owlet, we filtered 3,962 eBird and 20 iNaturalist records to a final count of 2,743 eBird and 15 iNaturalist records (total *n* = 2,758).

### 2.2 Data collection—climate data

We extracted the climate dataset available for four time periods, LIG (~120–140 Ka), LGM (~22 Ka), MDH (~6 Ka), and current (1960–1990) from <http://www.worldclim.org/> (Hijmans, Cameron, Parra, Jones, & Jarvis, 2005). Datasets for the LGM, MDH, and current time period were available at 2.5′ (around 5 km^2^); this being the highest resolution for the LGM and MDH datasets. The available dataset for the LIG was 30″ (around 1 km^2^) resolution (Otto‐Bliesner et al., 2006). Therefore, we scaled the LIG dataset to 2.5′. We used the LGM and MDH dataset from the Community Climate System Model (CCSM4) (Gent et al., 2011) following Fuentes‐Hurtado, Hof, and Jansson (2016). We clipped raster files of the bioclimatic variables at two extents to be used in the analysis, the Indian Subcontinent for the Indian owlets restricted to the Indian Subcontinent (5 N to 39.1 N and 55.1 E to 109.9 E) and Eurasia and parts of North Africa for Little Owl (0.71 N to 63.62 N and −20.08 E to 134.46 E). The geographical extent under modeling is a crucial factor in determining the accuracy of species distribution models (Barve et al., 2011; VanDerWal, Shoo, Graham, & Williams, 2009). Therefore, we used two different extents to capture the predictor range better.

### 2.3 Climatic niche models

We created bias files for all owlets to correct for sampling bias in modeling (Kramer‐Schadt et al., 2013). We used MaxEnt v 3.4.1 (Phillips, Anderson, & Schapire, 2006) for CNMs. We performed all the Pre‐ and Post‐MaxEnt data analyses in ArcGIS v10.1 (ESRI, 2011) and SDMToolbox (Brown, 2014) in ArcGIS. For CNMs, we followed Fuentes‐Hurtado et al. (2016) modeling protocol with modifications. We first performed a correlation analysis on all 19 bioclimatic predictors for the current time period to detect highly correlated (*r* > 0.8, *r* < −0.8) variables. To select the appropriate variables from pairs of highly correlated ones, all 19 variables were used in a MaxEnt run (replicate type = bootstraps, replicate runs = 50) and variables that contributed maximally in jackknifing runs were noted. For further analysis, we retained only those variables (from a correlated pair) that had high contributions in the MaxEnt output and were important considering the natural history of each owlet. This procedure has been used elsewhere (Carroll, 2010; Peterson & Robins, 2003). The selected variables for each species are shown in Table 1. We followed this procedure to fine‐tune the models and avoid overfitting (Lee‐Yaw et al., 2016; Radosavljevic & Anderson, 2014). The final MaxEnt models were run with 50 bootstrap iterations. We set the regularization parameter to 1.5 to avoid overfitting of data. To determine the robustness of the model in terms of Test and Training AUC values, we randomly picked 25% of points as test points. We performed backward‐time simulations by projecting CNM for the current period for each owlet at three time periods, MDH, LGM, and LIG. We used a 10th percentile logistic training presence threshold to convert continuous raster maps into binary maps to better visualize the change in the extent of climatic niche. The 10th percentile logistic threshold is a conservative estimator of predicted climatic niches and has been applied to avoid overfitting of models (Kumar & Stohlgren, 2009; Pearson, Raxworthy, Nakamura, & Townsend Peterson, 2007). The fossil data available on the study owls are scanty, mainly available for the Little Owl from Europe (Bedetti & Pavia, 2013; Mlikovsky, 2002; Pavia, Manegold, & Haarhoff, 2014); hence, validation of the past CNMs was not possible. Here, as with other CNM approaches, we assume that current species–climate relationships have been maintained in the past.

**Table 1:** Summary of the best‐fit climatic niche models (CNMs) for the current time period. Variables used are the same as for the past‐projections

Species	Variables used	Training AUC	Test AUC
Forest Owlet	BIO2, BIO5, BIO6, BIO9, BIO10, BIO11, BIO15, BIO18	0.996	0.994
Spotted Owlet	BIO3, BIO6, BIO7, BIO11, BIO12, BIO13, BIO15	0.879	0.876
Jungle Owlet	BIO2, BIO3, BIO4, BIO10, BIO11, BIO12, BIO13, BIO15	0.951	0.949
Little Owl	BIO1, BIO5, BIO10, BIO11, BIO14, BIO18, BIO19	0.926	0.924

Note

BIO1, Mean annual temperature; BIO2, Mean diurnal range; BIO3, Isothermality; BIO4, Temperature seasonality; BIO5, Maximum temperature of warmest month; BIO6, Minimum temperature of coldest month; BIO7, Temperature annual range; BIO9, Mean temperature of driest quarter; BIO10, Mean temperature of warmest quarter; BIO11, Mean temperature of coldest quarter; BIO12, Annual precipitation; BIO13, Precipitation of wettest month; BIO14, Precipitation of driest month; BIO15, Precipitation seasonality; BIO18, Precipitation of warmest quarter; BIO19, Precipitation of coldest quarter.

### 2.4 Post‐CNM analysis

We performed intersection and stability analyses on climatically suitable areas by superimposing suitable area polygons for a specific time period with another time period. We treated areas common to both the polygons (intersection analysis) as conserved areas (niche stable) and nonoverlapping areas as shift (contraction/expansion/displacement) in the climatic niche. We performed niche overlap analysis to compute *I *statistic (Warren, Glor, & Turelli, 2008) and niche breadth analysis to compute the B2 statistic (uncertainty index) (Nakazato, Warren, & Moyle, 2010) using ENMTools v1.4.4 (Warren, Glor, & Turelli, 2010) to explore niche overlap across time periods and species. The niche overlap index (*I* statistic) varies between 0 (no overlap) and 1 (complete overlap). The higher values in case of B2 index represent broader niche. Finally, we created a climatic heterogeneity layer for each time period using SDMToolbox in ArcGIS. The climatic heterogeneity information is in percentage (0–100), 0 signifying highly homogenous (stable) climate and a value of 100 indicating highly heterogeneous climate. Climatic heterogeneity information was extracted based on 1,000 random points generated for each suitable area polygon to test if the predicted climatically suitable areas of owlets fall in areas with higher climatic stability, expecting that suitable areas for the endemic Forest Owlet will be nested in climatically stable zones as compared to the widespread owlets. We performed one‐way ANOVA test on the climatic heterogeneity data to test for variation within a species across time periods and between species for each time period.

## 3 RESULTS

### 3.1 Climatically suitable areas and niche breadth of owlets

**Figure 2:**
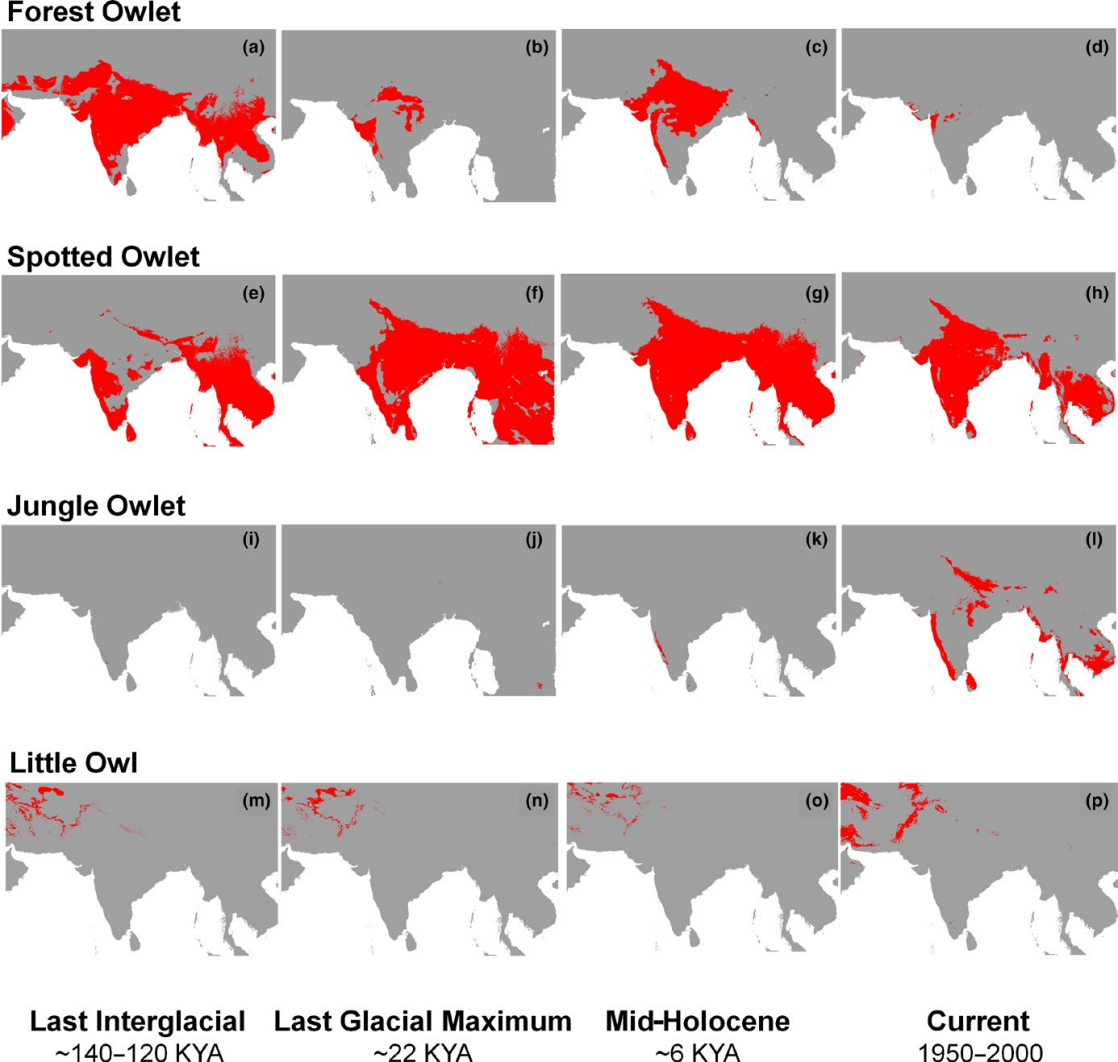
Binary maps of climatically suitable areas suggest that Quaternary climatic fluctuations affected all owlets differently. The red and gray colors indicate suitable and unsuitable areas, respectively. (a–d) The Forest Owlet maps; (e–h) The Spotted Owlet maps; (i–l) The Jungle Owlet maps; and (m–p) The Little Owl maps. The map of climatically suitable areas of the Little Owl is clipped to match the modeling extent used for other owlets for a comparative purpose

The models had a low false positive rate (model summaries in Table 1). Climatically suitable areas for the Forest Owlet underwent a cyclic reduction and expansion throughout the four time periods (Figure 2a–d, Table 2, Supporting Information Appendix S1: Figure S1.1.), with the maximum niche breadth and extent of suitable areas attained during the LIG and minimal during current time period. The suitable areas for the Forest Owlet in central India and the northern Western Ghats appeared to be conserved across time (Supporting Information Appendix S1: Figure S1.1.). Results for the Spotted Owlet indicate that climatically suitable areas for the species underwent expansion during the LGM and progressive reduction in MDH and current time periods (Figure 2e–h, Table 2, Supporting Information Appendix S1: Figure S1.2.). Climatically suitable areas for the Jungle Owlet showed a progressive expansion post‐LIG up to the current time period (Figure 2i–l, Table 2, Supporting Information Appendix S1: Figure S1.3.). Post‐LIG, during the LGM period climatically suitable areas for the Little Owl, reduced, but progressively expanded post‐LGM up to the current time period (Figure 2m–p, Table 2, Supporting Information Appendix S1: Figures S1.4. and S1.5.). We detected a southward shift during the LGM and northward expansion post‐LGM in climatically suitable areas for the Little Owl (Figure 2m, Table 2, Supporting Information Appendix S1: Figure S1.5.).

**Table 2:** Climatically suitable areas and niche breadth for owlets across four time periods

Species	Time period	Extent (km^2^)	Niche Breadth – B2 (*100)
Forest Owlet	Current	21,197	82.69
	MDH	486,051	93.4
	LGM	140,794	93.62
	LIG	1,261,067	94.76
Spotted Owlet	Current	941,649	93.55
	MDH	1,474,825	94
	LGM	1,714,729	94.58
	LIG	812,163	93.14
Jungle Owlet	Current	260,700	90.50
	MDH	6,297	85.37
	LGM	2,425	85.67
	LIG	478	85.51
Little Owl	Current	1,781,868	92.72
	MDH	289,030	82.1
	LGM	301,570	79.12
	LIG	360,646	78.54

Note

B2: Niche breadth in the range of 0 to 1 (low to high).

### 3.2 Climatic heterogeneity and niche overlap

**Figure 3:**
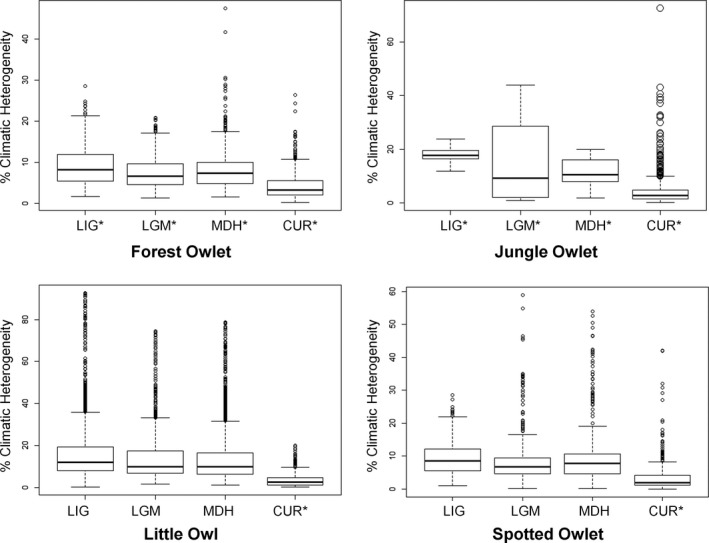
The species‐wise arrangement of the climatic heterogeneity values (ranges from 0—highly homogeneous to 100—highly heterogeneous) extracted from 1,000 random points selected from climatically suitable areas of the study owlets. CUR, Current; LGM, Last Glacial Maximum; LIG, Last Interglacial; MDH, Mid‐Holocene. * indicates that the values differ significantly (*p < *0.001, one‐way ANOVA test) from any other category

**Figure 4:**
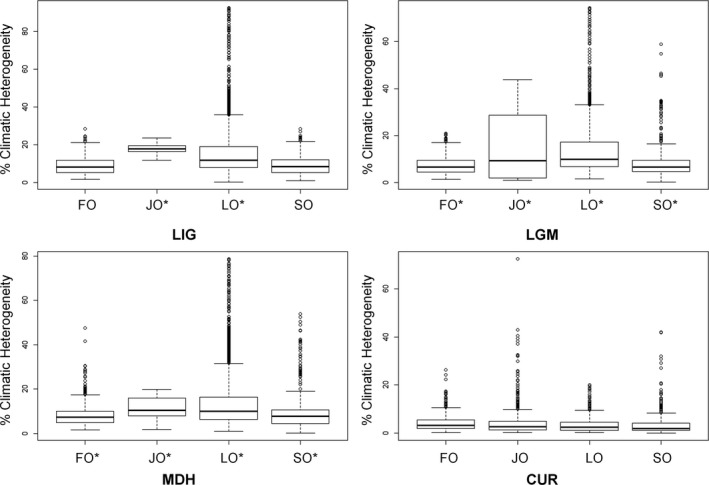
The time period‐wise arrangement of the climatic heterogeneity values (ranges from 0—highly homogeneous to 100—highly heterogeneous) extracted from 1,000 random points selected from climatically suitable areas of the study owlets. CUR: Current; FO: Forest Owlet; JO: Jungle Owlet; LGM: Last Glacial Maximum; LIG: Last Interglacial; LO: Little Owl; MDH: Mid‐Holocene; SO: Spotted Owlet. * indicates that the values differ significantly (*p < *0.001, one‐way ANOVA test) from any other category

We detected overlap (*I* = 0.62–0.98) in climatic niche of the Forest Owlet across the four time periods (Supporting Information Appendix S2: Table S2.1.). A similar pattern was seen for the Jungle Owlet (*I = *0.59–0.86) and Spotted Owlet (*I* = 0.94–0.98). For the Little Owl, niche overlap was the least (*I = *0.29–0.59) across the four time periods. When comparing overlap in niche between pairs of species for each time period, high overlap was observed between the Forest Owlet and Spotted Owlet (*I* > 0.9), except for the current time period (*I = *0.66). The Spotted Owlet, Jungle Owlet, and Little Owl showed high niche overlap only in the current time period (*I* > 0.8). The modeled climatically suitable areas of all the owlets were nested in climatically stable areas throughout the four time periods (Figures 3 and 4, Supporting Information Appendix S1: Figures S1.6. and S1.7., Appendix S2: Table S2.2.). In the current time period, all owlets occupied areas with higher climatic stability than in the past.

## 4 DISCUSSION

### 4.1 Quaternary climatic fluctuations and climatically suitable areas for owlets

Assuming that the owlets have tracked the climatically suitable areas predicted by our models, we detected variable responses of the four owlets to the Quaternary climatic fluctuations. The climatically suitable areas for the currently severely range‐restricted Endangered Forest Owlet showed distinct cycles of reduction and expansion; whereas suitable areas for other currently widespread owlets showed either an overall progressive expansion or reduction (Figure 2, Table 2). The change in climatically suitable areas for the Indian owlets might be a function of climate and climate‐mediated change in habitat, prey, and interactions among these species. Currently, the Forest Owlet is sympatric with Jungle Owlet and Spotted Owlet whereas the Jungle Owlet and Spotted Owlet overlap in occurrence in parts of their overall distributional ranges.

The niche breadth and extent of climatically suitable areas for the Forest Owlet were at its maximum in the LIG (Table 2), which was wetter and warmer than the pre‐industrial Holocene. During this time period, other Indian owlets had relatively constricted climatically suitable areas in the Indian Subcontinent. During the LIG, Jungle Owlet which is currently associated with forest and scrubland habitats and has a relatively younger divergence (1.8–0.1 Ma, Koparde et al., 2018), might have survived in the pockets of the south Western Ghats, especially in the eastern parts of the Western Ghats (Figure 2). Considering the LIG scenario (Figure 2), it appears that Jungle Owlet had an insignificant presence in Peninsular India and hence would have played a negligible role as a possible competitor to other Indian owlets at this time. Little Owl was widespread across the Palearctic in LIG and occupied relatively northward areas as compared to its climatically suitable areas during the LGM.

During the LGM, climatically suitable areas for the Forest Owlet showed a drastic reduction while for the Spotted Owlet they expanded. This is in tune with their known associations with forests and open habitats, respectively. In LGM, observations of drastic vegetation change in Indian Peninsula from moist rainforests (Prabhu et al., 2004; Sukumar, Suresh, & Ramesh, 1995) to tropical grasslands (Ray & Adams, 2001) indicate that the LGM climate possibly generated habitat suitable for Spotted Owlet. The climatically suitable areas for Jungle Owlet, however, showed a slight increase during LGM when compared to LIG, possibly occupying a diversity of habitats (moist to dry forests to scrublands). Climatically suitable areas for the Little Owl showed a southward shift during the LGM and a postglacial northward expansion (Figure 2, Supporting Information Appendix S1: Figure S1.5.). Climatically suitable areas for Little Owl during the LGM were not restricted to the European Southern Refugia, but widespread occupying a larger area than previously thought. Our results lend support to the Pellegrino et al., (2014) hypothesis of southward range shift of the Little Owl during the LGM.

The expansion in climatically suitable areas for Forest Owlet, during the MDH, could be due to prevailing climatic conditions that were comparable to LIG and when woodlands were widespread. In the case of Jungle Owlet, climatically suitable areas increased during the MDH and spread into the Western Ghats, overlapping with suitable areas for the other owlets. There is increasing evidence supporting multiple warm and cold climate cycles (Chauhan, 2002; Gupta, Anderson, & Overpeck, 2003; Randhawa, 1945; Sukumar, Ramesh, Pant, & Rajagopalan, 1993) and aridification (Ponton et al., 2012) in the tropics during the Holocene. In Early Holocene, there is evidence of changing vegetation in the Western Ghats (Kumaran et al., 2014; Srivastava, Pal, Aruche, Wani, & Sahrawat, 2015). Post‐MDH until the current time period such short‐term climatic fluctuations might have further impacted climatically suitable areas for the Indian owlets. The possible climate‐mediated vegetation changes post‐MDH and human‐mediated land‐use change postindustrialization might have impacted forest‐associated species such as Forest Owlet and Jungle Owlet, and open habitat associated species such as Spotted Owlet and Little Owl differently.

Although the owlets overlap in geographic distributions, there are few studies examining resource sharing among the species (Ishtiaq, 2000; Jathar & Rahmani, 2004; Mehta et al., 2018; Rasmussen & Ishtiaq, 1999; Yosef, Pande, Pawashe, Kasambe, & Mitchell, 2010). Interspecific interactions and resource use when factored into niche models could improve predictions (Araújo & Luoto, 2007; Wisz et al., 2013). Incorporating data on recent as well as paleoclimatic fluctuations generated for time periods not covered in this study are recommended, to obtain a more comprehensive picture of species responses to climate change.

### 4.2 Climatically suitable areas for owlets and climatic heterogeneity

Following the hypothesis of endemic species occupying climatically stable areas (Dynesius & Jansson, 2000; Jansson, 2003), over a gradient of range extents, we expected the following pattern: the Forest Owlet (forest‐associated species with the lowest EOO) would occupy climatically stable areas to a greater extent across all time periods, followed by the Jungle Owlet (associated with forest and scrubland), Spotted Owlet (associated with open habitat and human settlements), and Little Owl (associated with open habitat and human settlements but with the largest EOO). Our results showed that the predicted climatically suitable areas for all owlets were nested in climatically stable areas but to differing degrees in various time periods. For the Forest Owlet, climatically suitable areas were located within climatically stable areas during all four time periods, but for the Jungle Owlet and Spotted Owlet, this was not consistent across all time periods (Figures 3 and 4; Supporting Information Appendix S2: Table S2.4.). During the current time period, modeled suitable areas for all the owlets are within climatically stable areas (Figures 3 and 4) unlike during the MDH. The current nested geographical distributions of the owlets (Figure 1) could perhaps be explained by habitat tracking, presuming that the modeled suitable areas are good proxies for actual geographical distributions. The climate refugia for each owlet is different and needs to be mapped and projected considering future climate change for focused and effective conservation planning (Hannah et al., 2007).

### 4.3 Caveats and conclusion

There is no definitive way to empirically validate the past distribution models constructed for the study species and hence interpretations are presumptive. Nonavailability of fossil data for focal species makes it difficult to examine the accuracy of the past distribution models. The two central assumptions of the study are (a) climatically suitable area is a proxy for the geographical area occupied by a species and (b) the current species–climate relationships have been maintained in the past. We recommend validating the past distribution models with the help of fossil occurrence data whenever available. Apart from these major issues, the quality and accuracy of the predictor dataset and its projections are of concern (*reviewed in* Nogués‐Bravo, 2009).

Our results suggest that Quaternary climatic fluctuations might have played a significant role in shaping the present distribution of owlets. Such information can help in deciphering the biogeography of species, with varying habitat associations albeit with overlapping geographical distributions. Future research in this area should focus on more substantial datasets incorporating information on interspecific interactions and regional climate to understand the effect of Holocene climatic fluctuations on species.

## CONFLICT OF INTEREST

None declared.

## AUTHOR CONTRIBUTIONS

P.K., V.V.R., and S.M conceived the ideas; P.K. and P.M. collected the data; P.K. analyzed the data; and P.K., V.V.R., and S.M. led the writing.

## Data Availability

The climatic niche model output raster files can be accessed from Data Dryad Repository https://doi.org/10.5061/dryad.9fp97gk.
