# Supplementary material for: Quaternary climatic fluctuations and resulting climatically suitable areas for Eurasian owlets
Source: Ecol Evol. 2019 Mar 26;9(8):4864–74. doi: 10.1002/ece3.5086 (PMC6476768; doi:10.1002/ece3.5086)
Supplement: Supplementary file 1 [file ECE3-9-4864-s001.docx]

**Appendix S1 – Supplementary Figures**

**Quaternary Climatic Fluctuations and Resulting Climatically Suitable Areas for Eurasian Owlets**

**
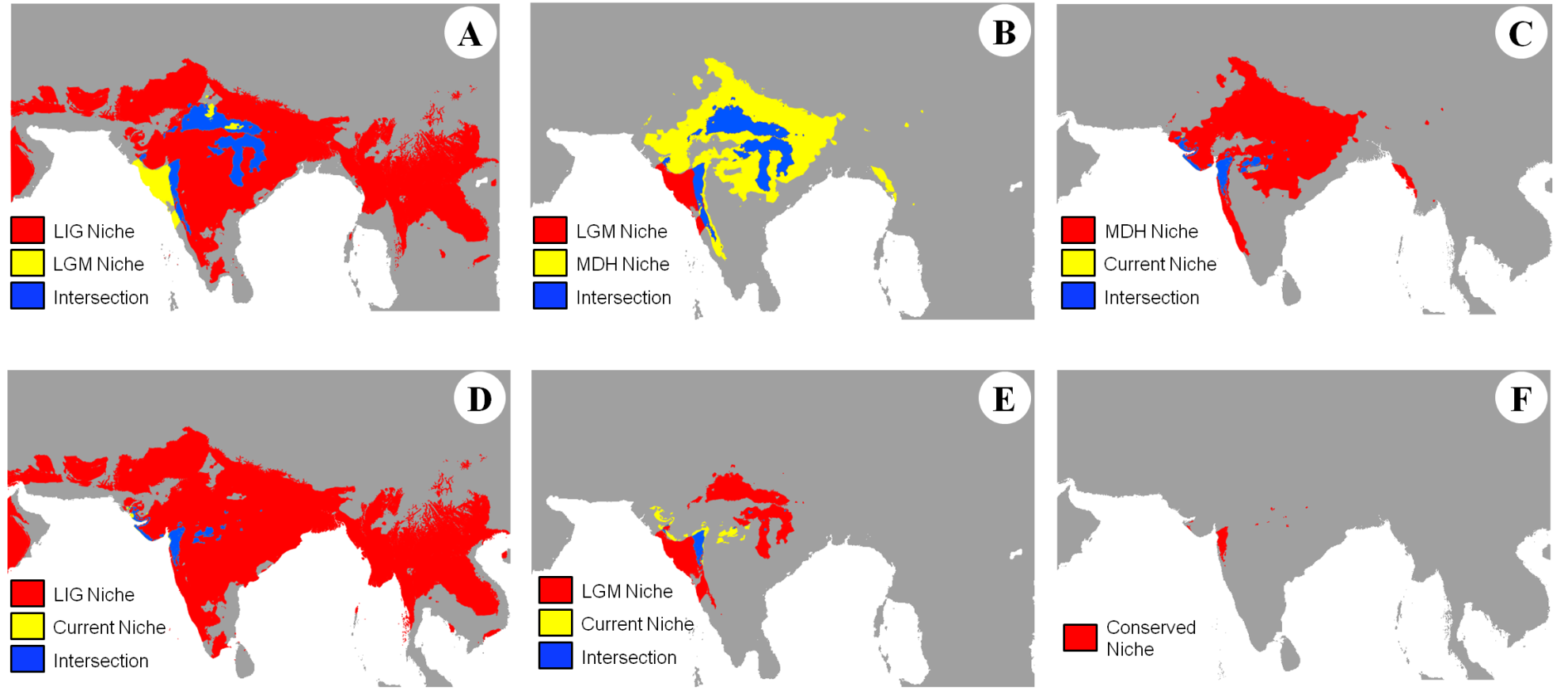
S1.1. Figure.** **Change in the climatically suitable areas for Forest Owlet across four time-periods.** Grey areas represent unsuitable areas. Climatically suitable areas are presented using colors. Each inset map contains color legend. **A:** Change between the LIG and LGM climatically suitable area; **B:** Change between the LGM and MDH climatically suitable area; **C:** Change between the MDH and current climatically suitable area; **D:** Change between the LIG and current climatically suitable area; **E:** Change between the LGM and current climatically suitable area; **F:** Conserved climatically suitable area across the four time periods. **LIG:** Last Interglacial, **LGM:** Last Glacial Maximum, **MDH:** Mid-Holocene.


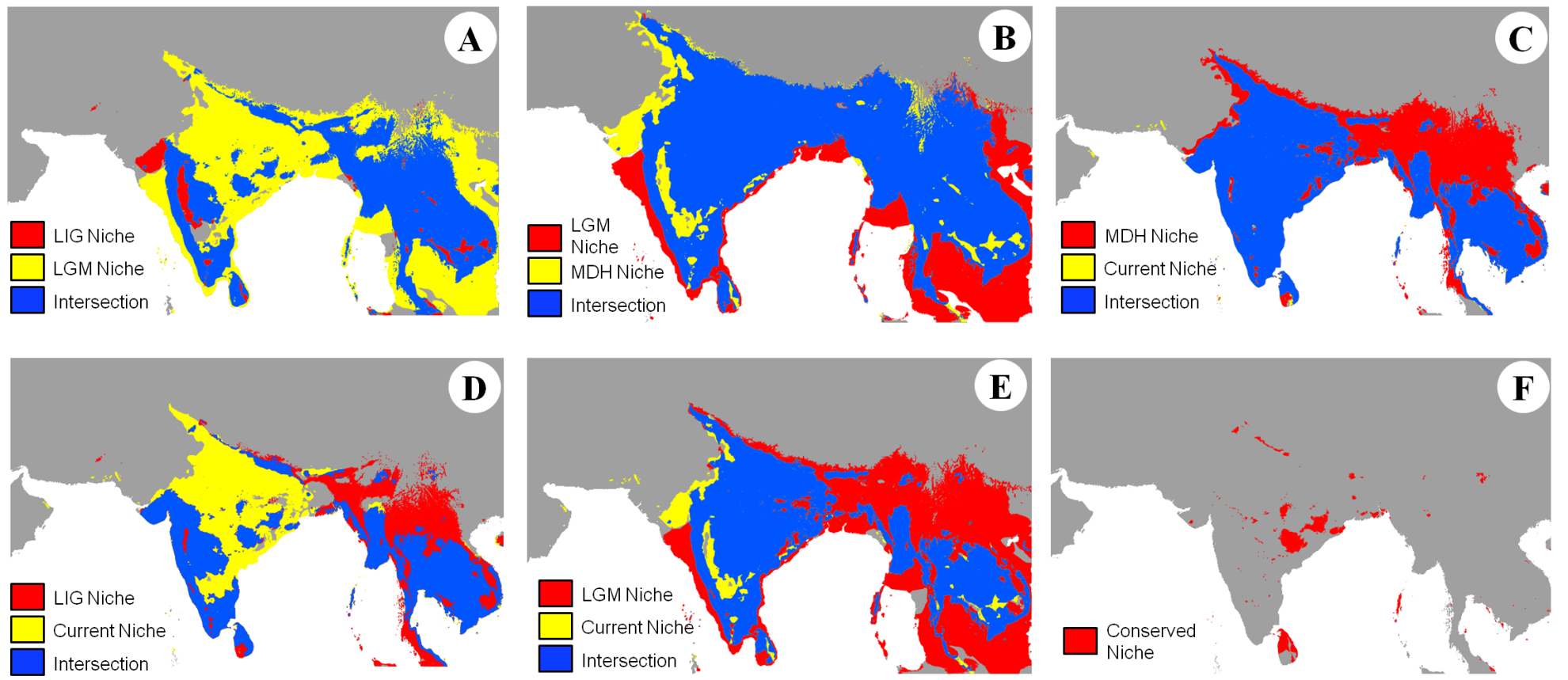


**S1.2. Figure.** **Change in the climatically suitable area for Spotted Owlet across four time-periods.** Grey areas represent unsuitable areas. Climatically suitable areas are presented using colors. Each inset map contains color legend. **A:** Change between the LIG and LGM climatically suitable area; **B:** Change between the LGM and MDH climatically suitable area; **C:** Change between the MDH and current climatically suitable area; **D:** Change between the LIG and current climatically suitable area; **E:** Change between the LGM and current climatically suitable area; **F:** Conserved climatically suitable area across the four time periods. **LIG:** Last Interglacial, **LGM:** Last Glacial Maximum, **MDH:** Mid-Holocene.


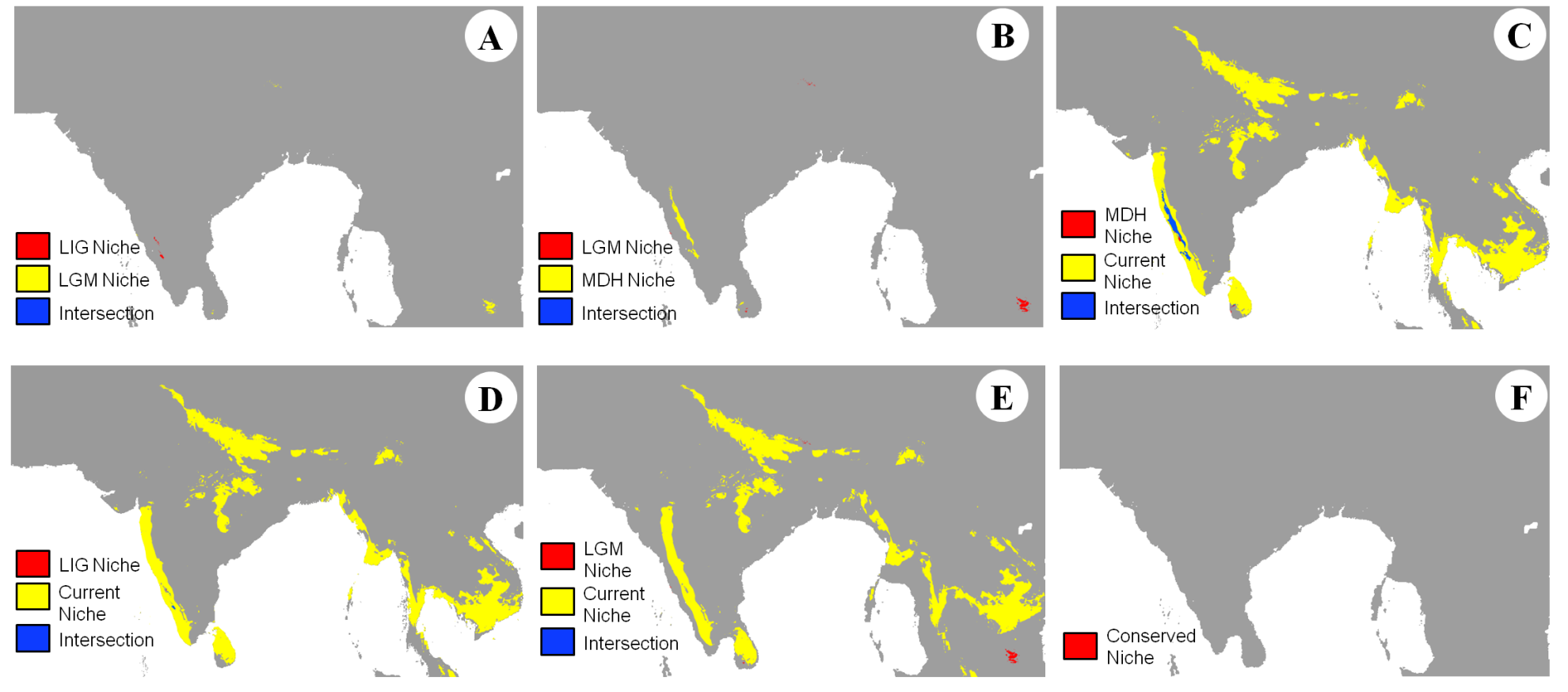


**S1.3. Figure.** **Change in the climatically suitable area for Jungle Owlet across four time-periods.** Grey areas represent unsuitable areas. Climatically suitable areas are presented using colors. Each inset map contains color legend. **A:** Change between the LIG and LGM climatically suitable area; **B:** Change between the LGM and MDH climatically suitable area; **C:** Change between the MDH and current climatically suitable area; **D:** Change between the LIG and current climatically suitable area; **E:** Change between the LGM and current climatically suitable area; **F:** Conserved climatically suitable area across the four time periods. **LIG:** Last Interglacial, **LGM:** Last Glacial Maximum, **MDH:** Mid-Holocene.

**
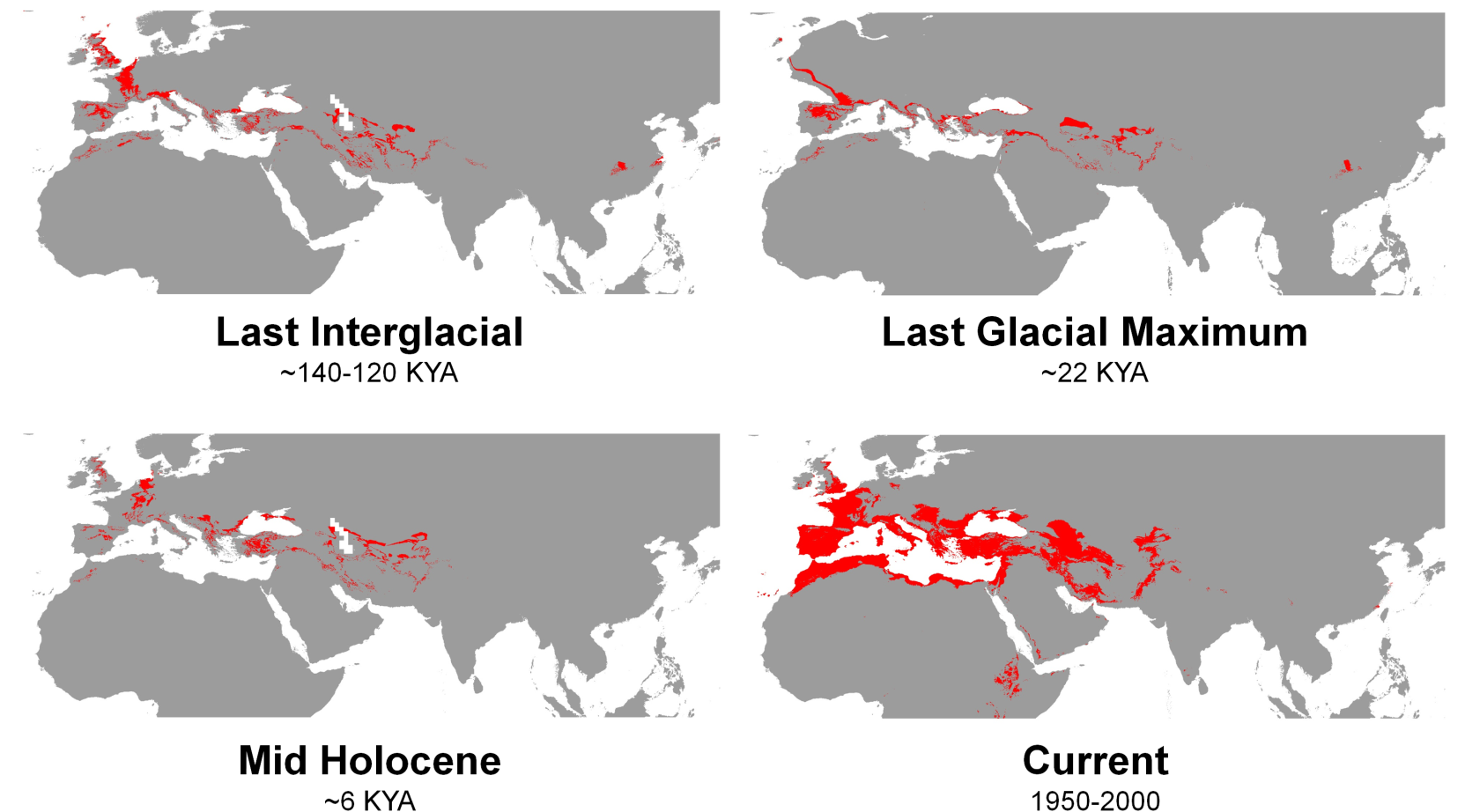
**

**S1.4. Figure. Binary maps of the climatically suitable areas for Little Owl.** The red and grey colors indicate suitable and unsuitable areas respectively.


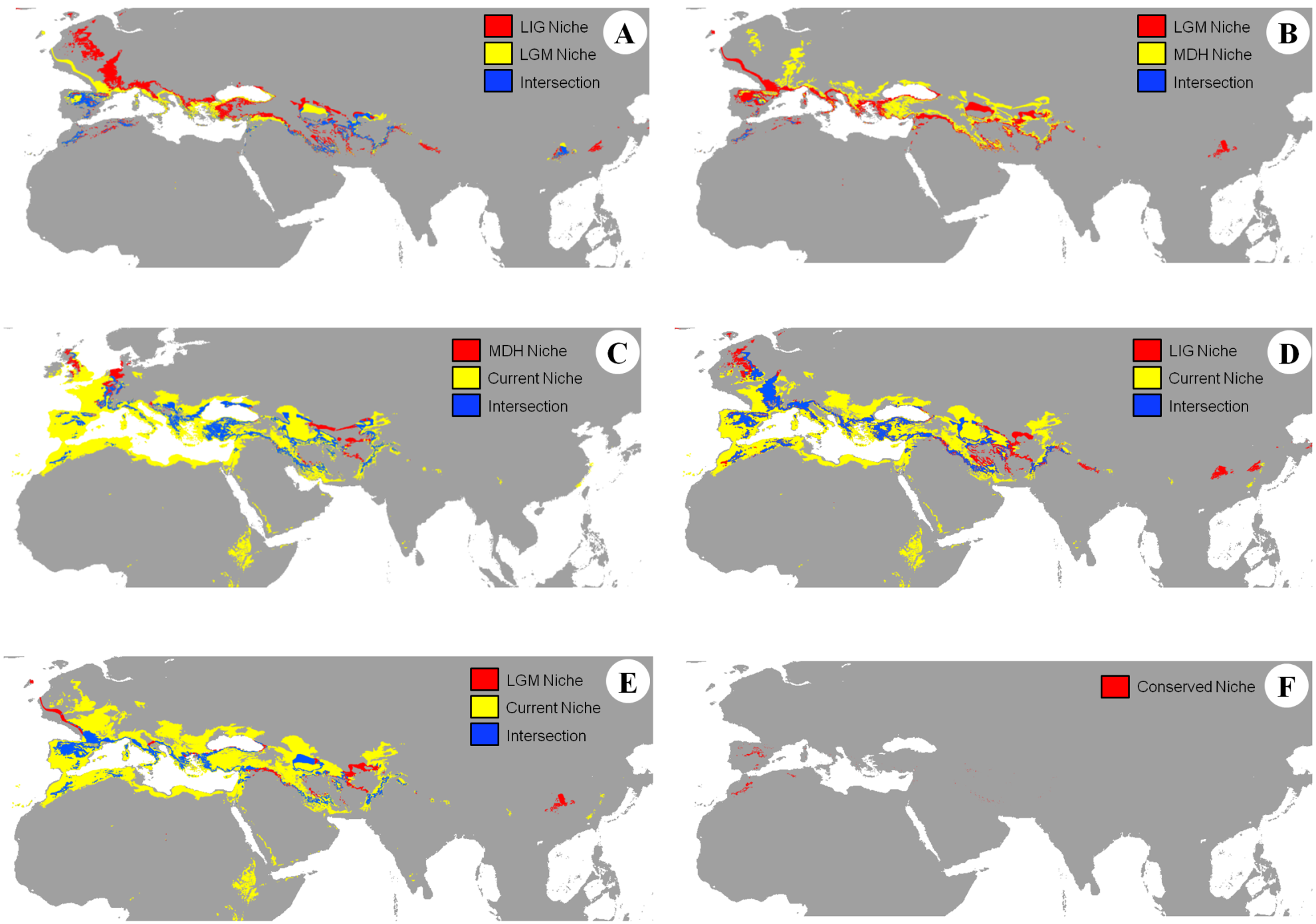


**S1.5. Figure.** **Change in the climatically suitable areas for Little Owl across four time-periods.** Grey areas represent unsuitable areas. Climatically suitable areas are presented using colors. Each inset map contains color legend. **A:** Change between the LIG and LGM climatically suitable area; **B:** Change between the LGM and MDH climatically suitable area; **C:** Change between the MDH and current climatically suitable area; **D:** Change between the LIG and current climatically suitable area; **E:** Change between the LGM and current climatically suitable area; **F:** Conserved climatically suitable area across the four time periods. **LIG:** Last Interglacial, **LGM:** Last Glacial Maximum, **MDH:** Mid-Holocene.


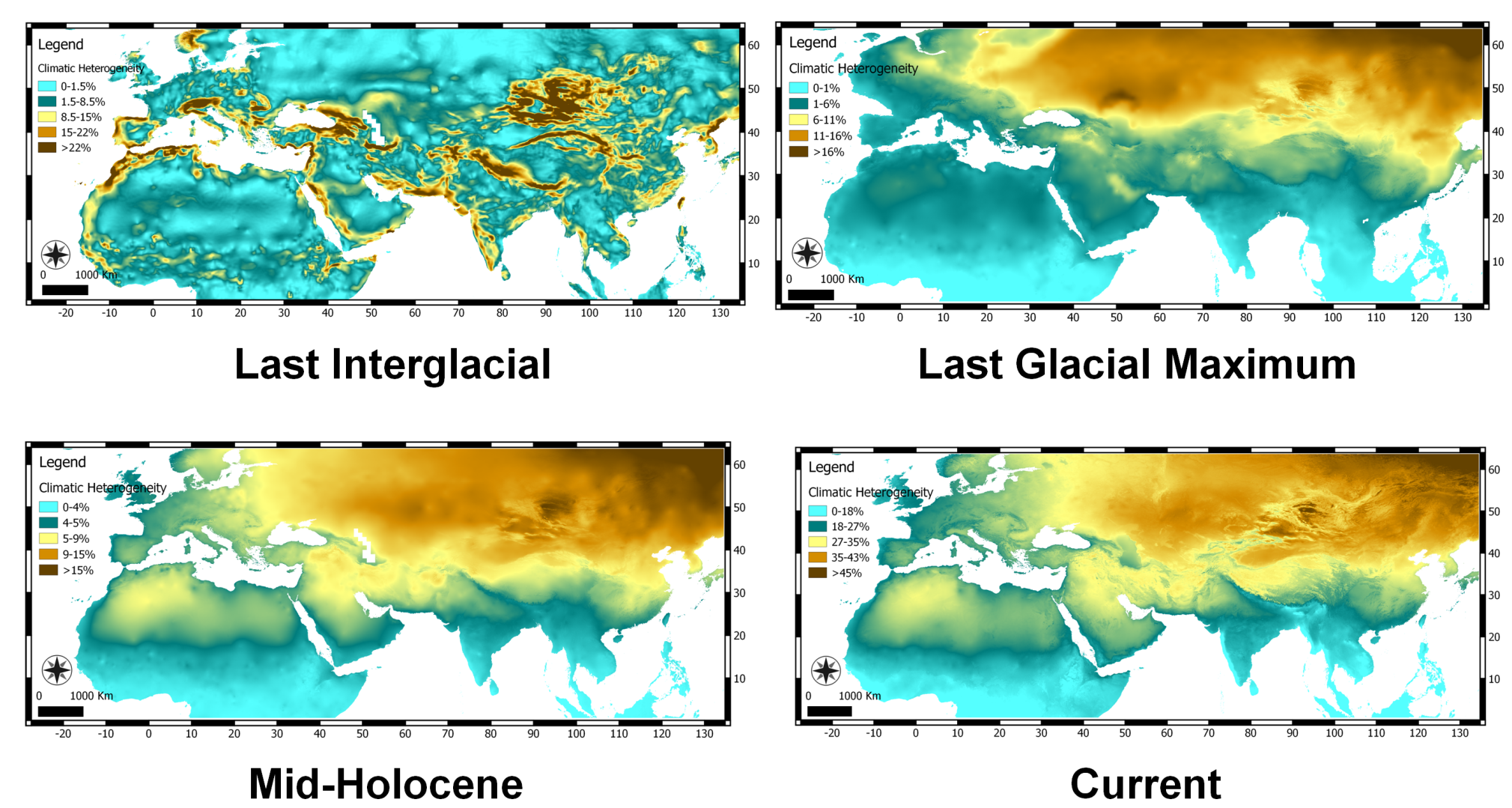


**S1.6. Figure.** **Climatic heterogeneity across four time periods.** Cyan color indicates climatically stable areas.

**
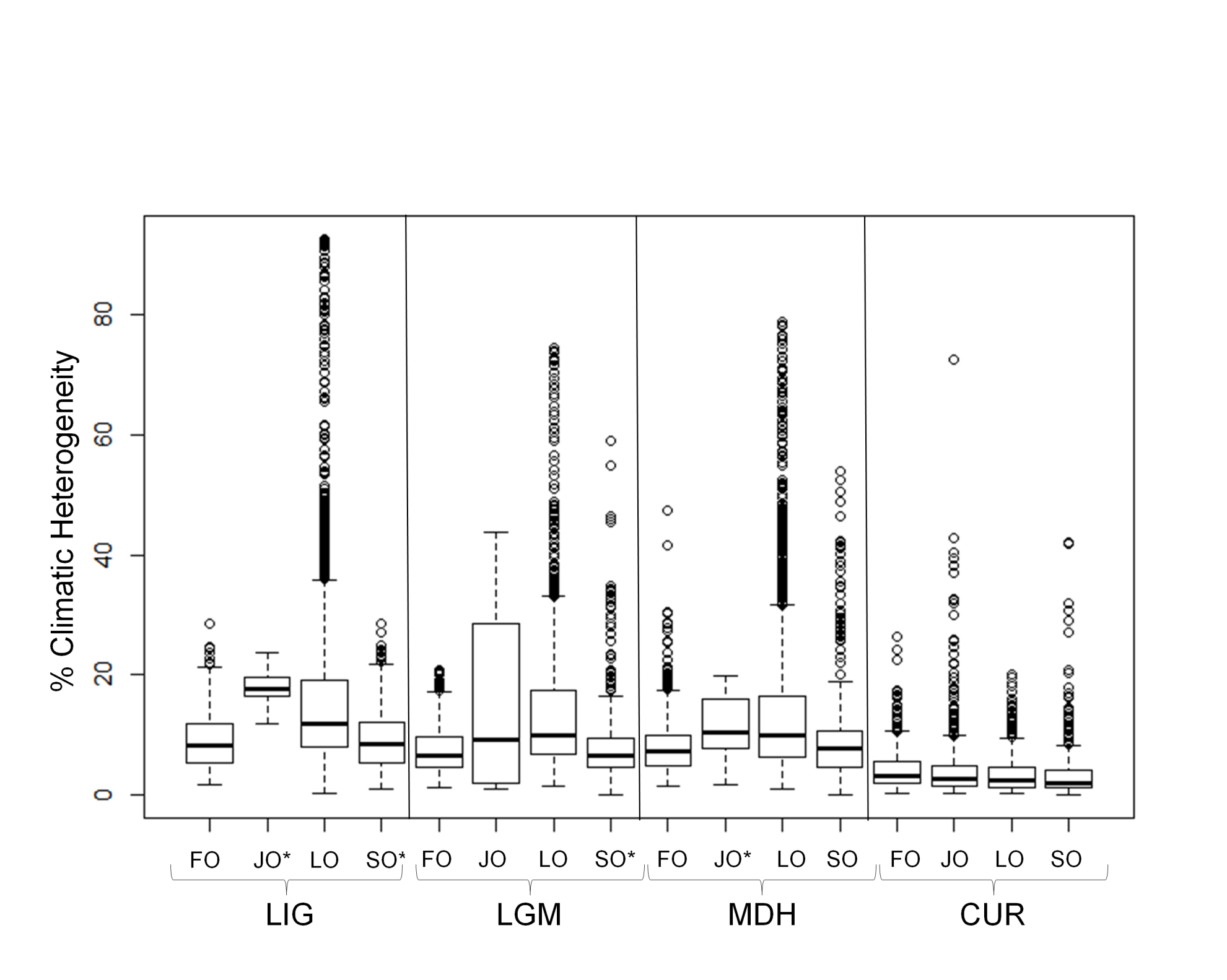
**

**S1.7. Figure.** **Climatic heterogeneity values extracted from 1000 random points selected from climatically suitable areas of the study owlets.** **LIG:** Last Interglacial, **LGM:** Last Glacial Maxima, **MDH:** Mid-Holocene, **CUR:** Current, **FO:** Forest Owlet, **SO:** Spotted Owlet, **LO:** Little Owl, **JO:** Jungle Owlet. *****indicates that the values differ significantly (*p* < 0.001) from any other species within the section (One-way ANOVA test).
