# Supplementary material for: Quaternary climatic fluctuations and resulting climatically suitable areas for Eurasian owlets
Source: Ecol Evol. 2019 Mar 26;9(8):4864–74. doi: 10.1002/ece3.5086 (PMC6476768; doi:10.1002/ece3.5086)
Supplement: Supplementary file 2 [file ECE3-9-4864-s002.docx]

**Appendix S2 – Supplementary Tables**

**Quaternary Climatic Fluctuations and Resulting Climatically Suitable Areas for Eurasian Owlets**

**S2.1. Table.** **Summary of *I* statistic of climatic niche overlap (0 = no overlap, 1 = total overlap) across time-periods.**

|  | | Jungle Owlet | | | | Spotted Owlet | | | | Little Owl | | | | Forest Owlet | | |
| --- | --- | --- | --- | --- | --- | --- | --- | --- | --- | --- | --- | --- | --- | --- | --- | --- |
|  |  | LIG | LGM | MDH | CUR | LIG | LGM | MDH | CUR | LIG | LGM | MDH | CUR | LIG | LGM | MDH |
| Jungle Owlet | LIG | - |  |  |  |  |  |  |  |  |  |  |  |  |  |  |
|  | LGM | 0.79 | - |  |  |  |  |  |  |  |  |  |  |  |  |  |
|  | MDH | 0.74 | 0.82 | - |  |  |  |  |  |  |  |  |  |  |  |  |
|  | CUR | 0.59 | 0.76 | 0.86 | - |  |  |  |  |  |  |  |  |  |  |  |
| Spotted Owlet | LIG | 0.52 | - | - | - |  |  |  |  |  |  |  |  |  |  |  |
|  | LGM | - | 0.7 | - | - | 0.95 |  |  |  |  |  |  |  |  |  |  |
|  | MDH | - | - | 0.77 | - | 0.95 | 0.98 |  |  |  |  |  |  |  |  |  |
|  | CUR | - | - | - | 0.89 | 0.94 | 0.95 | 0.97 |  |  |  |  |  |  |  |  |
| Little Owl | LIG | 0.43 | - | - | - | 0.59 | - | - | - |  |  |  |  |  |  |  |
|  | LGM | - | 0.5 | - | - | - | 0.49 | - | - | 0.55 |  |  |  |  |  |  |
|  | MDH | - | - | 0.63 | - | - | - | 0.72 | - | 0.45 | 0.29 |  |  |  |  |  |
|  | CUR | - | - | - | 0.87 | - | - | - | 0.92 | 0.5 | 0.53 | 0.59 |  |  |  |  |
| Forest Owlet | LIG | 0.38 | - | - | - | 0.91 | - | - | - | 0.52 | - | - | - |  |  |  |
|  | LGM | - | 0.57 | - | - | - | 0.93 | - | - | - | 0.36 | - | - | 0.92 |  |  |
|  | MDH | - | - | 0.67 | - | - | - | 0.94 | - | - | - | 0.56 | - | 0.93 | 0.96 |  |
|  | CUR | - | - | - | 0.57 | - | - | - | 0.66 | - | - | - | 0.57 | 0.62 | 0.73 | 0.68 |

**LIG:** Last Interglacial, **LGM:** Last Glacial Maxima, **MDH:** Mid Holocene, **CUR:** Current, - indicates not applicable.

**S2.2. Table. The summary statistic for the climatic heterogeneity values for each owlet.**

| Species | Time period | Average | Standard Deviation | Range |
| --- | --- | --- | --- | --- |
| Forest Owlet | LIG | 9.2 | 4.9 | 1.7 - 28.5 |
|  | LGM | 7.5 | 4 | 1.3 - 20.8 |
|  | MDH | 8.5 | 5.4 | 1.5 - 47.5 |
|  | CUR | 4.3 | 3.5 | 0.2 - 26.3 |
| Spotted Owlet | LIG | 9.4 | 5.1 | 1.1 - 28.6 |
|  | LGM | 8.4 | 7.1 | 0.1 - 59 |
|  | MDH | 9.3 | 8.3 | 0.1 - 54 |
|  | CUR | 3.4 | 4.6 | 0 - 42 |
| Little Owl | LIG | 15.1 | 11.1 | 0.3 - 93 |
|  | LGM | 13.4 | 10.2 | 1.7 - 74.5 |
|  | MDH | 13.3 | 11 | 1 - 79 |
|  | CUR | 3.5 | 3.4 | 0.2 - 20 |
| Jungle Owlet | LIG | 18 | 1.9 | 11.9 - 23.9 |
|  | LGM | 15.4 | 13.7 | 1 - 43.9 |
|  | MDH | 11 | 5 | 1.9 - 20 |
|  | CUR | 4.5 | 6 | 0.2 - 72.5 |

**LIG:** Last Interglacial, **LGM:** Last Glacial Maximum, **MDH:** Mid Holocene, **CUR:** Current.
